# Safety and Visual Outcome of Visian Toric ICL Implantation after Corneal Collagen Cross-Linking in Keratoconus: Up to 2 Years of Follow-Up

**DOI:** 10.1155/2015/514834

**Published:** 2015-03-19

**Authors:** Rafic Antonios, Ali Dirani, Ali Fadlallah, Elias Chelala, Adib Hamade, Carole Cherfane, Elias Jarade

**Affiliations:** ^1^Faculty of Medicine, American University of Beirut, Beirut, Lebanon; ^2^Beirut Eye Specialist Hospital, P.O. Box 116-5311, Al-Mathaf Square, Beirut, Lebanon; ^3^Faculty of Medicine Beirut, Saint-Joseph University, Beirut, Lebanon; ^4^Mediclinic, Dubai Mall, Dubai, UAE

## Abstract

*Purpose*. To evaluate the long-term safety and clinical outcome of phakic Visian toric implantable collamer lens (ICL) insertion after corneal collagen cross-linking (CXL) in progressive keratoconus. *Methods*. This was a retrospective study of 30 eyes (19 patients), with progressive keratoconus, who underwent sequential CXL followed by Visian toric ICL implantation after 6 months. *Results*. At baseline, 6 eyes had stage I, 14 eyes stage II, and 10 eyes stage III keratoconus graded by Amsler-Krumeich classification. At 6 months after CXL, only *K* (steep) and *K* (max) decreased significantly from baseline, with no change in visual acuity or refraction. Flattening in keratometric readings was stable thereafter. There was significant improvement in mean uncorrected distance visual acuity (1.57 ± 0.56 to 0.17 ± 0.06 logMAR, *P* < 0.001) and mean corrected distance visual acuity (0.17 ± 0.08 to 0.11 ± 0.05 logMAR, *P* < 0.001) at 12 months after ICL implantation that was maintained at the 2-year follow-up. Mean cylinder power and mean spherical equivalent (SE) also decreased significantly after ICL implantation. A small hyperopic shift in SE (+0.25 D) was observed at 2 years that did not alter visual outcomes. *Conclusions*. Visian toric ICL implantation following CXL is an effective option for improving visual acuity in patients with keratoconus up to 2 years.

## 1. Introduction

Keratoconus is a progressive noninflammatory thinning disorder of the cornea leading to a decrease in visual acuity as a result of myopia and irregular astigmatism [1, 2]. Corneal collagen cross-linking (CXL) can effectively halt the progression of the disease [2], but visual acuity following CXL remains poor. In patients intolerant to rigid gas permeable contact lenses after corneal CXL, additional interventions are often necessary to improve their vision [1, 2].

Many visual rehabilitation options are available to manage keratoconus including intracorneal ring segment implantation (ICRS), phakic intraocular lenses (pIOL), and photorefractive keratectomy (PRK) and all can be combined with CXL [3–6]. In patients with poor best-corrected visual acuity ICRS implantation is performed [7–9]. The PRK is used to correct mild refraction error [3, 4], while the pIOL's are used to correct moderate to severe ametropia in patient with good best-corrected visual acuity [5, 6].

Our study group recently published the 6-month data on the safety and efficacy of CXL followed by insertion of a phakic toric implantable collamer lens (ICL) (Visian Toric V4 ICL; STAAR Surgical, Monrovia, CA) in the posterior chamber for correction of myopia and astigmatism in patients with keratoconus [9]. In this paper, we report the long-term safety and efficacy of sequential CXL, then ICL implantation, separated by 6 months, in a larger cohort of patients with moderate to severe keratoconus with moderate to severe myopia and astigmatism, and good best-corrected visual acuity.

## 2. Methods

### 2.1. Patient Selection

This was a retrospective study of patients with keratoconus who underwent sequential CXL-ICL procedure between December 2010 and March 2012 at the Beirut Eye Specialist Hospital (BESH), Beirut, Lebanon. This study was approved by the Institutional Review Board at BESH and complied with the declaration of Helsinki. All patients signed an informed consent prior to treatment and all surgical procedures were performed by one surgeon (E.J).

Patients treated according to Dr. Jarade's protocol [9] were included if they had a preoperative best-correct visual acuity better than 20/40, were hard contact lens intolerant (defined as a comfortable wearing time of less than 8 hours per day), had an endothelial count >2,200 cells/mm^2^ (Noncon Robo, Konan Medical), had history of progressive keratoconus in one or both eyes (defined as an increase in maximum keratometry of 1.00 diopter (D) or more in 1 year and/or the need for new contact lens fitting more than once in the previous 2 years), and did not have any corneal surgery (including PRK and ICRS) before or after the CXL and ICL implantation. Patients were considered eligible for ICL implantation after CXL only if the keratoconus was considered stable (defined as subjective refractions [5, 9, 10] within ±0.50 D of spherical equivalent at 4 and 6 months postoperatively and was most of the time equivalent to the refraction prior to CXL).

The exclusion criteria for enrollment in this study (those who could not undergo the CXL and phakic IOL procedures consecutively) were central corneal thickness of less than 450 *μ*m (measured by optical pachymetry (Pentacam; Oculus Optikgerate GmbH, Wetzlar, Germany)), mean *K* reading >56.00, endothelial cell count of less than 2,000 cells/mm^2^ measured on the central part of the cornea by specular microscopy, anterior chamber depth of <2.8 mm from endothelium to anterior capsule measured by Pentacam (Oculus Optikgerate GmbH, Wetzlar, Germany), corneal opacification or scars, history of keratitis (any form), peripheral marginal degeneration, previous corneal and/or intraocular surgeries, and autoimmune and/or connective tissue disease. The central corneal thickness limit of 450 *μ*m would account for around 400 *μ*m of remaining stromal thickness after removal of the epithelium [11], which is considered as the safety thickness for the residual stroma to avoid endothelial cell damage during the CXL procedure [12].

The criteria for diagnosing keratoconus were based on a combination of computed slit-scanning videokeratography of the anterior and posterior corneal surfaces, keratometric readings, and corneal pachymetry [13–16]. Keratoconus was classified, according to the Amsler-Krumeich criteria, into four stages based on corneal power, thickness, transparency, and astigmatism [17].

Contact lens use was discontinued for at least 3 weeks for rigid lenses and 1 week for soft lenses prior to any ophthalmic examination, investigation, and treatment. The preoperative and postoperative screening consisted of a complete ophthalmic examination. It included uncorrected distance visual acuity (UDVA), corrected distance visual acuity (CDVA), manifest and cycloplegic refractions, anterior and posterior segments evaluation with dilated fundus examination, and keratometric evaluation. Since the autorefractometer results of refraction are not always accurate in keratoconus and after both CXL procedures, all refractions were based on refined refraction using trial lenses, and the axis of astigmatism was chosen according to the best visual acuity obtained while rotating the astigmatism trial axis [9].

Follow-up examinations were scheduled at baseline and at 1, 3, 6, and 12 months and every 6 months thereafter.

### 2.2. Cross-Linking Procedure

The eye to be treated was anesthetized by applying proparacaine hydrochloride 0.5% drops on three occasions at 5-minute intervals. After positioning the patient under the operating microscope, an eyelid speculum was inserted and the central 9 mm corneal epithelium was removed with a blunt spatula. A mixed riboflavin 0.1%–20% dextran solution was instilled every 5 minutes until the riboflavin penetrated the cornea (i.e., approximately 30 minutes). The ultraviolet lamp (UV-X illumination system, version 1000; IROC AG, Zurich, Switzerland) was then focused on the apex of the cornea at a distance of 5 cm for a total of 30 minutes, providing a radiant energy of 3.0 ± 0.3 mW/cm^2^. The required irradiance of 3.0 mW/cm^2^ was calibrated prior to each treatment using an ultraviolet A meter (LaserMate-Q; LASER 2000, Wessling, Germany). During ultraviolet A administration, riboflavin drops were applied to the cornea every 5 minutes. The cross-linking procedure adopted in our study is in accordance with the standard “Dresden” protocol, which has been shown to result in absorption limited to the anterior two-thirds (200–400 *μ*m) of the stroma as demonstrated by stress-strain measurements, thermomechanical measurements, and swelling studies [12].

Thinnest and central corneal thickness were continuously monitored (Sonogage Pachymeter; Sonogage, Inc., Cleveland, OH) to ensure that neither of the two parameters dropped below 400 *μ*m. After treatment, the eye surface was washed with balanced salt solution and two drops of gatifloxacin 0.3% were instilled, followed by placement of a bandage soft contact lens. Postoperatively, patients received acetaminophen 500 mg twice daily for 3 days, one drop of gatifloxacin 0.3% six times daily for 7 days with one drop of tobramycin-dexamethasone 0.1% four times daily for 10 days, and one drop of loteprednol 0.5% five times daily, slowly tapered over 5 weeks. The bandage soft contact lens was removed on postoperative day 4 and the eye examined by slit-lamp microscopy to confirm complete corneal epithelialization. Complete assessment was performed 1 and 6 months postoperatively and included UDVA, CDVA, refraction, and anterior/posterior topography. No further progression of keratoconus was noted in any eyes throughout the 6 months of follow-up period.

### 2.3. ICL Insertion Procedure

The implantation of the toric ICL was performed at least 6 months after CXL. ICL power was calculated using the software provided by the manufacturer. Emmetropia was selected as the target refraction. The appropriate ICL size was determined based on the horizontal white-to-white distance measured manually with a caliper, and the anterior chamber depth was measured with the Pentacam. A minor clinical adjustment of anterior chamber depth was performed by subtracting no more than 0.2 mm whenever corneal anterior bulging was advanced. Regarding the inaccuracy of the autorefractometer in predicting the *K*-reading in many keratoconus cases and to obtain accurate ICL choice using the online ICL calculator software, adjustment of extreme values of *K* readings obtained by autorefractometer was performed by attenuating the *K*-reading values to reflect the magnitude of astigmatism obtained by manifest refraction and the chosen axis of astigmatism was always the axis obtained by manifest refraction.

Laser iridotomy was performed 1 week preoperatively. The pupil was dilated with cyclopentolate and phenylephrine drops, instilled 30 minutes prior to surgery, and the horizontal axis was marked by the surgeon with the patient upright to control for cyclotorsion. General anesthesia was administered to all patients. A 3.2 mm clear corneal tunnel incision was performed in the horizontal temporal meridian (regardless of the astigmatism axis). The anterior chamber was filled with sodium hyaluronate 1%. The ICL was inserted in the posterior chamber through the incision using the injector cartridge supplied by the manufacturer. After the ICL was gently positioned in the sulcus with the axis properly aligned, the remaining viscoelastic material was completely washed out of the anterior chamber with balanced salt solution and a miotic agent was instilled. No intraoperative complications were encountered. Tobramycin-dexamethasone 0.1% eye drops were used four times a day for 10 days and then slowly tapered over 3 weeks.

### 2.4. Statistical Analysis

SPSS version 20.0 was used for data management and analyses. Descriptive statistics were reported as mean and standard deviation for continuous variables. Repeated-measures analysis with the Bonferroni test for post hoc analysis and the Wilcoxon Signed Rank test were computed. *P* value < 0.05 was considered to be statistically significant.

## 3. Results

The study included 30 eyes of 19 patients, among those 13 males and 6 females. Mean age was 30.44 ± 8.14 years (range: 20 to 45 years). Mean follow-up was 16 ± 5.75 months; all patients (100%) had complete follow-up from baseline up to 12 months after ICL implantation; only 10 (33%) eyes of 10 patients had 24 months of follow-up. The Visian toric ICL was implanted in all eyes; 11 patients underwent bilateral implantation while the remaining 8 patients had unilateral ICL implantation. Preoperative mean spherical power was −8.37 ± 3.89 D (range: −20.5 to −4 D) and mean cylindrical power 2.95 ± 1.40 D (range: 1 to 5.25 D). According to the Amsler-Krumeich classification, 6 eyes had stage I, 14 eyes had stage II, and 10 eyes had stage III keratoconus at baseline. Among the eyes that completed the 24 months of follow-up after ICL implantation, 6/10 had stage I, 2/10 had stage II, and 3/10 had stage III keratoconus at baseline. All eyes had an endothelial cell count greater than 2,200 cells/mm_2_. The mean central corneal thickness was 479 ± 24 *μ*m.

### 3.1. Refractive Outcome

The preoperative values were compared to values starting 6 months after CXL, because visual acuity and corneal keratometry vary significantly in the first few months after CXL.

**Figure 1:**
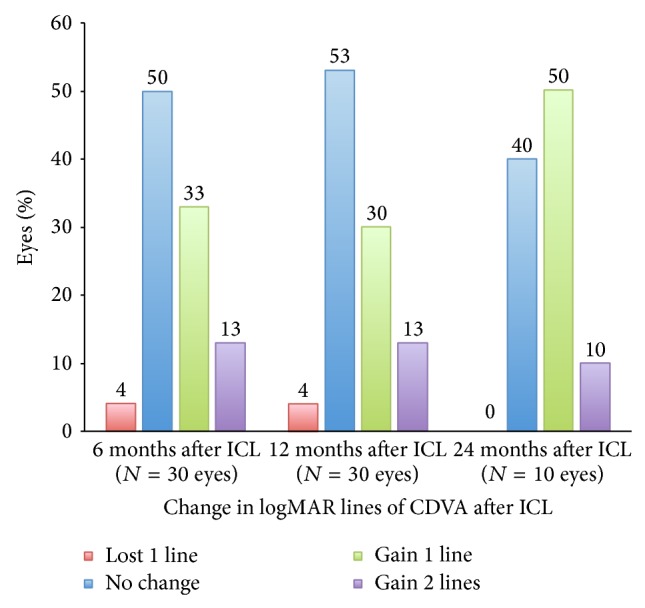
Change in corrected distance visual acuity (CDVA) following toric implantable collamer lens implantation (ICL).

According to Table 1, both UDVA and CDVA values at 6 months after CXL did not differ from baseline (*P* = 1.000 and 0.231, resp.). At 6 months after ICL implantation, there was significant improvement in mean UDVA from 1.57 logMAR to 0.17 logMAR (*P* < 0.001) and mild improvement in CDVA from 0.17 logMAR to 0.11 logMAR (*P* < 0.001). Both CDVA and UDVA remained stable thereafter up to 24 months (Tables 1 and 2). No eye lost 2 or more lines in CDVA in the study (Figure 1). At 12 months, 43% (13 of 30) of eyes gained ≥1 line in CDVA, and in the smaller subset of eyes with 24 months follow-up 60% of eyes gained ≥1 line in CDVA. Overall, 60% (18 of 30) and 50% (5 of 10) of eyes had UDVA of 20/30 or better 12 months and 24 months after ICL implantation, respectively.

**Figure 2:**
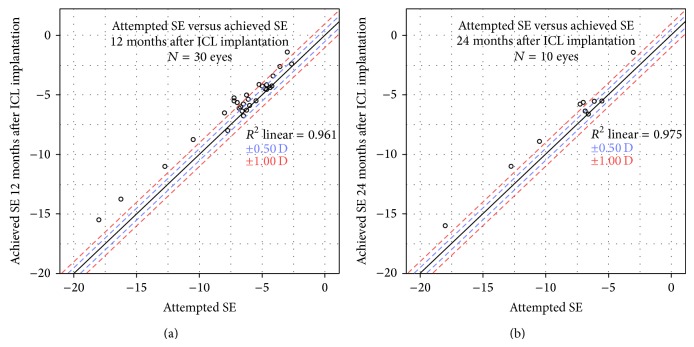
Attempted and achieved spherical equivalent (SE) correction at (a) 12 months and (b) 24 months after implantable collamer lens (ICL) implantation, respectively.

At 6 months after CXL, the small changes in SE and the spherical component of refraction were not significant from baseline (*P* = 0.611 and 1.000, resp.), unlike the mean change of 0.21 D in cylindrical component (*P* = 0.012) (Table 1). However, the changes in SE, sphere power and cylindrical power at 6 months after ICL implantation were all clinically and statistically significant from baseline and their values remained relatively stable up to 12 months (Table 1). However, in the smaller subset of 10 eyes with 24 months of follow-up (Table 2), small hyperopic shifts of 0.25 D in SE (*P* = 0.012) and 0.20 D in spherical power (*P* = 0.005) were noted after 6 months after ICL visit. Overall, 63.3% and 40% of eyes were within ±1.0 D SE at 12 and 24 months after ICL implantation, respectively (Figure 2).

All keratometric values showed a gradual decrease after CXL, up to the 24 months of follow-up. According to Table 1, the decrease in mean *K* (flat) from baseline became statistically significant 6 months after ICL implantation, while the decreases in mean *K* (steep) and mean *K* (max) from baseline were statistically significant starting 6 months after CXL.

**Figure 3:**
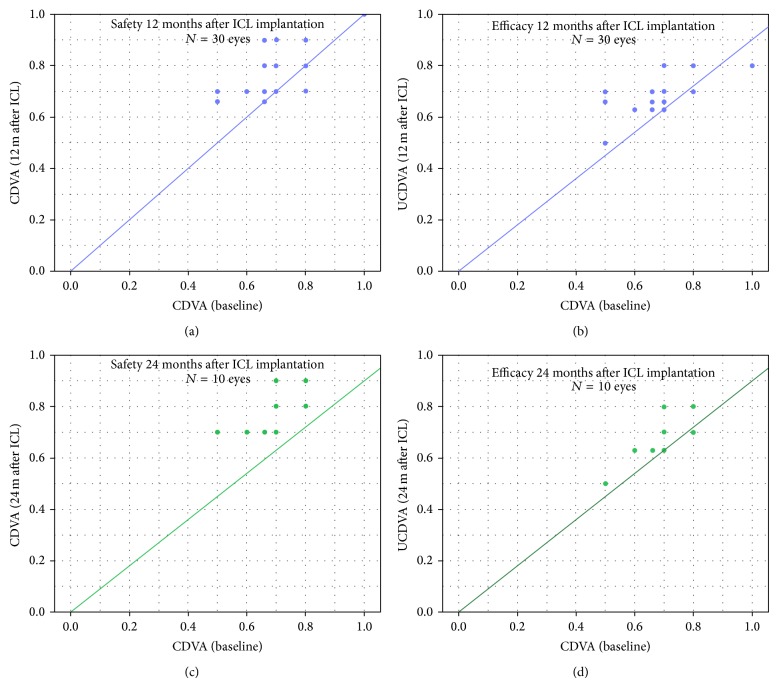
Safety and efficacy graphs comparing preoperative corrected distance visual acuity (CDVA) and uncorrected distance visual acuity (UDVA) 12 months (a, b) and 24 months (c, d) after ICL.

Overall, the safety index = [mean postoperative CDVA (logMAR)/mean preoperative CDVA (logMAR)] at 12 months and 24 months after ICL implantation was 0.73 ± 0.29 and 0.72 ± 0.25, respectively. The efficacy index = [mean postoperative UDVA (logMAR)/mean preoperative CDVA (logMAR)] at 12 months and 24 months after ICL implantation was 1.03 ± 0.26 and 1.04 ± 0.26, respectively (Figure 3).

### 3.2. Complications

All epithelial defects healed within 4 days after CXL. In this study, none of the patients had infectious keratitis, lens rotation, vaulting problem, cataract formation, pigment dispersion, or pupillary block. Also, none had development of clinically significant haze at any of the follow-up periods. There was, however, a transient increase of intraocular pressure that was observed in most patients during the first week after ICL implantation that was controlled with topical drops.

## 4. Discussion

Providing optimal refractive and vision results to patients with progressive keratoconus remains challenging to the refractive surgeon. While corneal collagen cross-linking (CXL) can halt progressive disease [2], patients with high refractive error and poor vision at baseline would remain so, after CXL, even without keratoconus progression [10, 18, 19]. Therefore, CXL is used to set the stage for other interventions to be performed. Management after CXL is tailored according to the patient's best-corrected visual acuity and refractive status. In patients with good-best corrected visual acuity and high residual refractive error after CXL, pIOL implantation provides adequate correction of ametropia [9]. Several types of toric pIOL were reported to be effective and safe in eyes with keratoconus, but only a handful of studies have evaluated their use following a CXL procedure [6, 9, 10, 18, 20–24].

The Visian toric ICL has demonstrated good efficacy and safety profiles for the correction of high ametropia in patients without keratoconus [25–32]. In our previous study [9], toric ICL implantation 6 months after CXL was proven to be an effective and safe method of improving visual acuity and refraction in selected eyes with moderate to severe keratoconus. In this paper, we assess the long-term (up to 24 months) safety and efficacy of that same procedure in 30 eyes with mild-to-moderate progressive keratoconus.

Stability of keratoconus following CXL in preparation for ICL implantation has been previously defined using stability of refraction data [5, 9, 10]. As such, ICL implantation was performed 6 months after CXL, since most patients had a stable visual acuity and manifest refraction by 4 months. The *K*-reading values however showed gradual flattening after CXL throughout the study. This flattening was not significant enough to alter the mean SE manifest refraction at the time of ICL implantation, or the outcome of the ICL procedure at 12 months. In the small subset of 10 eyes with 24 months of follow-up, the small hyperopic shift in SE might have resulted from the continuous flattening in *K* readings; however, the change did not affect vision. The continuous flattening in *K* readings and its effect on SE is most likely due to the effect of CXL [33]. It is unlikely that the 3.2 mm clear corneal incision at the time of ICL implantation (surgically induced astigmatism) would have contributed to the change in SE; the incisions were placed at the temporal site according to the surgeon's preference, regardless of the axis of manifest astigmatism. Another possible yet unlikely culprit of the change in SE is the rotation of the toric Visian ICL with loss in the refractive corrective effect [5]. Although possible, the effect of a rotation on refraction and visual acuity would have been uncovered earlier, and all our patients were happy with the end-result. After stabilization of keratoconus with CXL and ICL implantation, 60% (18 of 30) and 50% (5 of 10) of eyes had UDVA of 20/30 or better at 12 months and 24 months, respectively. Results of our study compare favorably to other reports in terms of gain in UDVA and CDVA [9, 10, 21], as reflected by the safety and efficacy indices. In our study, the slight myopic SE refraction at post-ICL implantation was related to 2 factors. First, there is no way to customize the ICL to exactly fit the patient's refraction, and in most cases we had to use what was available (undershoot the target refraction of plano). Second, one patient had a high refractive power that exceeds the capacity of the ICL (which is limited to −18.0 D of manifest refraction at the eyeglasses plane). However, all our patients were satisfied with the resulting vision.

Only 2 other studies have evaluated the safety and efficacy of Visian toric ICL following CXL [6, 10]. Both Kymionis et al. and Shafik Shaheen et al. evaluated the outcomes of Visian toric ICL implantation 12 months following CXL. Kymionis et al. [6] in a case report published encouraging results of this procedure; at 3 months, UDVA improved from counting fingers to 20/40 and CDVA improved from 20/100 to 20/30. Shafik Shaheen et al. [10], in a case series of 16 eyes with early-stage (undefined) keratoconus, showed a favorable outcome in terms of visual acuity and SE at 3 years of follow-up; mean CDVA improved from 20/35 to 20/22, mean UDVA improving to 20/23 and mean SE improving from −8.5 D to −0.25 D. In our previous study on mild to severe keratoconus [9], the 6-month results revealed that mean CDVA improved from 0.15 logMAR to 0.12 logMAR, mean UDVA decreased from 1.67 logMAR to 0.15 logMAR, and mean SE decreased to −0.89 D with no complication.

Other types of pIOLs implanted after CXL have also been evaluated. Izquierdo Jr et al. [18] employed the iris-fixated Artiflex phakic IOL (Ophtec, USA) in 11 eyes with progressive keratoconus. Results were favorable in terms of visual acuity, sphere, and cylinder at 12 months. Güell et al. [5] employed the toric Artiflex/Artisan phakic IOL in 17 keratoconic eyes; at 24 months, 14 eyes were within ±0.50 D of the attempted SE correction and 13 eyes were within ±1.00 D of the attempted cylinder correction.

ICL implantation after CXL depends on the stability of keratoconus (both refraction and keratometry) since progression would lead to refractive changes and drop of visual acuity [34]. A continuous flattening in *K* readings after CXL occurred in our study with no significant effect on SE, UDVA, nor CDVA at 1 year; in the smaller subset of 10 eyes a statistically but nonclinically significant change in SE was observed following CXL at the 2-year follow-up, but both UDVA and CDVA were not affected. Although we do believe that a longer time interval would possibly show a greater change in keratometry, we are still uncertain whether an equivalent amount of change in refractive error would accompany this flattening, possibly related to the altered biomechanics of cross-linked corneas. As demonstrated in our results, the change in keratometry did not significantly alter the SE and more importantly did not alter the UDVA and CDVA. The small hyperopic shift observed in our study deserves further investigation with long-term studies to assess its long-term impact on vision, but it does not warrant delaying ICL implantation. The continuous flattening effect of CXL with the accompanying risk for a hyperopic shift can last more than 2 years [35, 36]; therefore, targeting mild undercorrection rather than delaying the ICL implantation for 12 months would improve predictability and may be a solution. Moreover, implanting an ICL at 6 months as opposed to 12 months offers the patient the benefit of earlier functional visual recovery.

In conclusion, the results of toric ICL implantation 6 months after CXL at 1 year and at 2 years compare to the outcomes at 6 months in a previous study; it is an effective and safe method of improving visual acuity and refraction in keratoconus eyes with high myopia and astigmatism and good best corrected visual acuity.

## Figures and Tables

**Table 1:** Refractive data at baseline, 6 months after CXL, and 1 and 6 months after Visian toric ICL implantation for keratoconus (*N* = 30 eyes).

Parameter (mean ± SD)	Preoperatively	6 M after CXL [^a^ *P* value^*^]	6 M after ICL [^b^ *P* value^*^]	12 M after ICL [^c^ *P* value^*^]
UDVA (log⁡MAR)	1.57 ± 0.50	1.57 ± 0.56 [1.000]	0.17 ± 0.06 [<0.001]	0.17 ± 0.06 [1.000]
CDVA (log⁡MAR)	0.17 ± 0.08	0.15 ± 0.06 [0.231]	0.11 ± 0.05 [<0.001]	0.11 ± 0.05 [1.000]
Sphere (D)	−8.37 ± 3.89	−8.18 ± 3.64 [0.611]	−1.38 ± 0.96 [<0.001]	−1.36 ± 0.94 [1.000]
Cylinder (D)	2.95 ± 1.40	2.74 ± 1.33 [0.012]	1.12 ± 0.68 [<0.001]	1.03 ± 0.60 [0.094]
SE (D)	−6.96 ± 3.68	−6.81 ± 3.48 [1.000]	−0.86 ± 0.86 [<0.001]	−0.83 ± 0.76 [1.000]
*K* (flat) (D)	46.52 ± 3.72	46.23 ± 3.21 [0.588]	45.95 ± 3.79 [0.007]	45.94 ± 3.79 [1.000]
*K* (steep) (D)	50.49 ± 4.42	49.55 ± 4.18 [<0.001]	49.03 ± 4.61 [<0.001]	48.98 ± 4.65 [0.587]
*K* (max) (D)	53.08 ± 5.17	52.01 ± 4.87 [<0.001]	51.55 ± 4.78 [<0.001]	51.55 ± 4.75 [1.000]

CXL: corneal collagen cross-linking; ICL: implantable collamer lens; SD: standard deviation; UDVA: uncorrected distance visual acuity; CDVA: corrected distance visual acuity; D: diopters; *K*: keratometry values.

^*^ *P* value derived from post hoc analysis.

^a^Comparing 6 months after CXL to preoperative value.

^b^Comparing 6 months after ICL to preoperative value.

^c^Comparing 12 months after ICL to 6 months after ICL value.

**Table 2:** Complete case analysis of 10 eyes with 24 months of follow-up (*N* = 10 eyes).

Parameter (mean ± SD)	Preoperatively	6 M after CXL	6 M after ICL	12 M after ICL [^a^ *P* value′]	24 M after ICL [^b^ *P* value′]
UDVA (log⁡MAR)	1.75 ± 0.56	1.84 ± 0.69	0.17 ± 0.07	0.17 ± 0.06 [0.317]	0.17 ± 0.07 [0.317]
CDVA (log⁡MAR)	0.17 ± 0.07	0.15 ± 0.05	0.12 ± 0.05	0.12 ± 0.05 [1.000]	0.12 ± 0.05 [1.000]
Sphere (D)	−9.60 ±4.69	−9.55 ± 4.67	−1.83 ± 0.93	−1.75 ± 0.98 [0.317]	−1.63 ± 0.95 [0.005]
Cylinder (D)	2.55 ± 1.35	2.38 ± 1.29	1.15 ± 0.64	1.00 ± 0.59 [0.034]	1.05 ± 0.55 [0.102]
SE (D)	−8.32 ± 4.33	−8.36 ± 4.31	−1.34 ± 0.86	−1.17 ± 0.78 [0.027]	−1.09 ± 0.75 [0.012]
*K* (flat) (D)	45.57 ± 4.13	45.59 ± 3.38	44.97 ± 3.74	44.99 ± 3.76 [0.610]	44.93 ± 3.77 [0.131]
*K* (steep) (D)	49.17 ± 4.37	48.07 ± 4.42	47.31 ± 4.29	47.25 ± 4.35 [0.256]	47.29 ± 4.37 [0.581]
*K* (max) (D)	51.16 ± 4.57	50.30 ± 4.55	49.82 ± 4.36	49.80 ± 4.34 [0.715]	49.75 ± 4.35 [0.019]

CXL: corneal collagen cross-linking; ICL: implantable collamer lens; SD: standard deviation; UDVA: uncorrected distance visual acuity; CDVA: corrected distance visual acuity; D: diopters; *K*: keratometry values.

′*P* value derived from Wilcoxon Signed Rank test.

^a^Comparing 12 months after ICL to 6 months after ICL.

^b^Comparing 24 months after ICL to 6 months after ICL.
